# Diaqua­bis­(l-lactato)magnesium

**DOI:** 10.1107/S1600536812028723

**Published:** 2012-06-30

**Authors:** Hong-lin Zhu, Ling Jin

**Affiliations:** a Crystal Engineering Division, Center of Applied Solid State Chemistry Research, Ningbo University, Ningbo, Zhejiang 315211, People’s Republic of China

## Abstract

In the title compound, [Mg(C_3_H_4_O_3_)_2_(H_2_O)_2_], the Mg^2+^ cation is six-coordinated by four O atoms from two lactate anions and two aqua ligands, completing an MgO_6_ distorted octa­hedral geometry. The complex mol­ecules are bridged by O—H⋯O hydrogen-bonding inter­actions into helical chains parallel to the *a* axis, which are linked by further O—H⋯O inter­actions, forming a three-dimensional supra­molecular architecture.

## Related literature

For related compounds, see: Carballo *et al.* (2007 ▶); Chen *et al.* (2000 ▶); Qiu *et al.* (2010 ▶); Zeng *et al.* (2010 ▶).
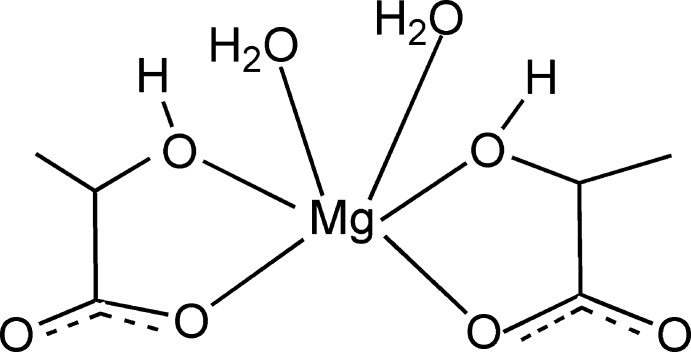


## Experimental

### 

#### Crystal data

[Mg(C_3_H_4_O_3_)_2_(H_2_O)_2_]
*M* *_r_* = 238.48
Orthorhombic, 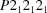

*a* = 6.0525 (12) Å
*b* = 11.919 (2) Å
*c* = 14.526 (3) Å
*V* = 1047.9 (4) Å^3^
*Z* = 4
Mo *K*α radiation
μ = 0.19 mm^−1^
*T* = 293 K
0.28 × 0.20 × 0.16 mm

#### Data collection

Rigaku R-AXIS RAPID diffractometer
Absorption correction: multi-scan (*ABSCOR*; Higashi, 1995 ▶) *T*_min_ = 0.955, *T*_max_ = 0.970
10148 measured reflections
1401 independent reflections
1208 reflections with *I* > 2σ(*I*)
*R*_int_ = 0.033

#### Refinement

*R*[*F* ^2^ > 2σ(*F* ^2^)] = 0.029
*wR*(*F* ^2^) = 0.074
*S* = 1.14
1401 reflections
154 parameters
8 restraints
H atoms treated by a mixture of independent and constrained refinement
Δρ_max_ = 0.25 e Å^−3^
Δρ_min_ = −0.27 e Å^−3^

### 

Data collection: *RAPID-AUTO* (Rigaku, 1998 ▶); cell refinement: *RAPID-AUTO*; data reduction: *CrystalStructure* (Rigaku/MSC, 2004 ▶); program(s) used to solve structure: *SHELXS97* (Sheldrick, 2008 ▶); program(s) used to refine structure: *SHELXL97* (Sheldrick, 2008 ▶); molecular graphics: *SHELXTL* (Sheldrick, 2008 ▶); software used to prepare material for publication: *SHELXL97*.

## Supplementary Material

Crystal structure: contains datablock(s) global, I. DOI: 10.1107/S1600536812028723/aa2062sup1.cif

Structure factors: contains datablock(s) I. DOI: 10.1107/S1600536812028723/aa2062Isup2.hkl

Additional supplementary materials:  crystallographic information; 3D view; checkCIF report

## Figures and Tables

**Table 1:** Hydrogen-bond geometry (Å, °)

*D*—H⋯*A*	*D*—H	H⋯*A*	*D*⋯*A*	*D*—H⋯*A*
O3—H3*D*⋯O5^i^	0.84	1.83	2.668 (2)	178
O6—H6*D*⋯O2^ii^	0.82	1.85	2.666 (2)	174
O7—H7*A*⋯O2^iii^	0.83	1.94	2.768 (2)	171
O7—H7*B*⋯O5^iv^	0.83	1.85	2.678 (2)	177
O8—H8*A*⋯O1^iii^	0.83	2.12	2.933 (2)	167
O8—H8*B*⋯O4^i^	0.83	1.94	2.765 (2)	168

Symmetry codes: (i) 
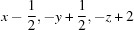
; (ii) 
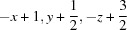
; (iii) 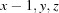; (iv) 
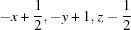
.

**Fig. 1.**
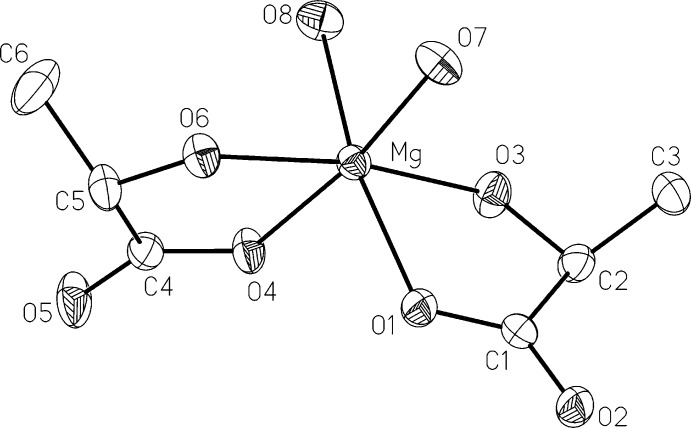
The content of asymmetric unit showing the atomic numbering and 45% probability dispalcement ellipsoids.

**Fig. 2.**
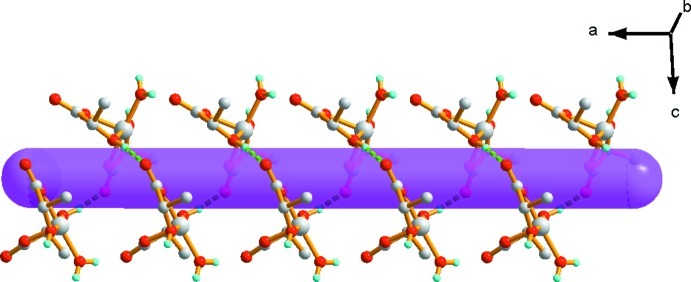
The one-dimensional helical chain.

**Fig. 3.**
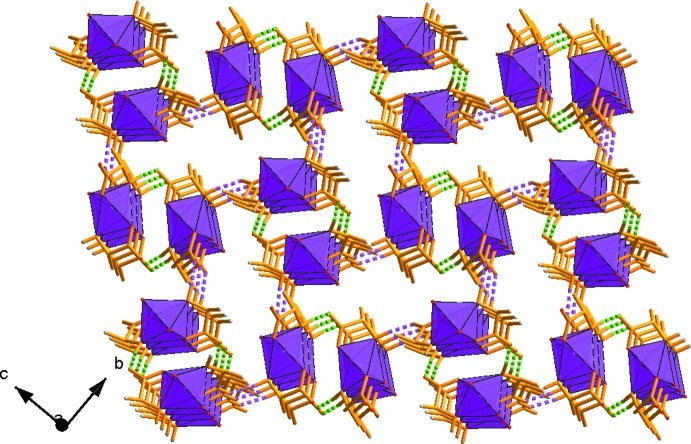
The three-dimensional supramolcular architecture in the title compound.

## supplementary crystallographic information

### Comment

In the past decades, more attention have been paid to design and rational synthesis of coordination polymers based on self-assemblies of metal ions with lactic acid. The crystal structures of a number of metal lactates and their complexes have been reported (Carballo *et al.*, 2007; Chen *et al.*, 2000; Qiu *et al.*, 2010; Zeng *et al.*, 2010).

In the asymmetric unit of the the title compound, Mg(H_2_O)_2_(C_3_H_4_O_3_)_2_, the Mg^2+^ cation is chelated by two lactate anions bound through the carboxylate and hydroxyl groups. The other two sites are occupied by water molecules forming MgO_6_ distorted octahedral geometry (Fig. 1). The complexes greatly favor strong hydrogen bonding in the crystalline state. The hydroxyl group O3—H3D of the lactate ligand forms a hydrogen bond with the carboxylate atom O5^i^ of a symmetry-related lactate anion (Table 1). This hydrogen bonding interaction leads to assemble the complexes into one-dimensional helical chain parallel to the *a* axis (Fig. 2). Other H-bonds link chains forming the three-dimensional architecture (Fig. 3).

### Experimental

Boric acid (1 mmol) and *L*-lactic acid (1 mmol) were dissolved in 10 ml distilled water. Magnesium hydroxide (0.5 mmol) was then added. The resulting suspension was stirred for 30 min and subsequently the white insoluble solid was filtered out. The colourless filtrate was finally kept at room temperature. After slow evaporation for two months colourless block-like crystals of the title complex were obtained. According to X-ray structure determination, boric acid did not participate in the complex formation.

### Refinement

H atoms bonded to C atoms were placed in geometrically calculated position and were refined using a riding model, with *U*_iso_(H) = 1.2 *U*_eq_(C). H atoms attached to O atoms were found in a difference Fourier synthesis and were refined with the O—H distance restrained to 0.83 (2) Å and *U*_iso_(H) = 1.2 *U*eq(O).

### Crystal data

**Table:**

[Mg(C_3_H_4_O_3_)_2_(H_2_O)_2_]	*F*(000) = 504
*M**_r_* = 238.48	*D*_x_ = 1.512 Mg m^−^^3^
Orthorhombic, *P*2_1_2_1_2_1_	Mo *K*α radiation, λ = 0.71073 Å
Hall symbol: P 2ac 2ab	Cell parameters from 8276 reflections
*a* = 6.0525 (12) Å	θ = 3.3–27.4°
*b* = 11.919 (2) Å	µ = 0.19 mm^−^^1^
*c* = 14.526 (3) Å	*T* = 293 K
*V* = 1047.9 (4) Å^3^	Block, colourless
*Z* = 4	0.28 × 0.20 × 0.16 mm

### Data collection

**Table:**

Rigaku R-AXIS RAPID diffractometer	1401 independent reflections
Radiation source: fine-focus sealed tube	1208 reflections with *I* > 2σ(*I*)
Graphite monochromator	*R*_int_ = 0.033
Detector resolution: 0 pixels mm^-1^	θ_max_ = 27.4°, θ_min_ = 3.3°
ω scans	*h* = −7→7
Absorption correction: multi-scan (*ABSCOR*; Higashi, 1995)	*k* = −15→15
*T*_min_ = 0.955, *T*_max_ = 0.970	*l* = −18→18
10148 measured reflections	

### Refinement

**Table:**

Refinement on *F*^2^	Primary atom site location: structure-invariant direct methods
Least-squares matrix: full	Secondary atom site location: difference Fourier map
*R*[*F*^2^ > 2σ(*F*^2^)] = 0.029	Hydrogen site location: inferred from neighbouring sites
*wR*(*F*^2^) = 0.074	H atoms treated by a mixture of independent and constrained refinement
*S* = 1.14	*w* = 1/[σ^2^(*F*_o_^2^) + (0.0268*P*)^2^ + 0.4768*P*] where *P* = (*F*_o_^2^ + 2*F*_c_^2^)/3
1401 reflections	(Δ/σ)_max_ < 0.001
154 parameters	Δρ_max_ = 0.25 e Å^−^^3^
8 restraints	Δρ_min_ = −0.27 e Å^−^^3^

### Special details

**Table:**

Geometry. All e.s.d.'s (except the e.s.d. in the dihedral angle between two l.s. planes) are estimated using the full covariance matrix. The cell e.s.d.'s are taken into account individually in the estimation of e.s.d.'s in distances, angles and torsion angles; correlations between e.s.d.'s in cell parameters are only used when they are defined by crystal symmetry. An approximate (isotropic) treatment of cell e.s.d.'s is used for estimating e.s.d.'s involving l.s. planes.
Refinement. Refinement of *F*^2^ against ALL reflections. The weighted *R*-factor *wR* and goodness of fit *S* are based on *F*^2^, conventional *R*-factors *R* are based on *F*, with *F* set to zero for negative *F*^2^. The threshold expression of *F*^2^ > σ(*F*^2^) is used only for calculating *R*-factors(gt) *etc*. and is not relevant to the choice of reflections for refinement. *R*-factors based on *F*^2^ are statistically about twice as large as those based on *F*, and *R*- factors based on ALL data will be even larger.

### Fractional atomic coordinates and isotropic or equivalent isotropic displacement parameters (Å^2^)

**Table:**

	*x*	*y*	*z*	*U*_iso_*/*U*_eq_	
Mg	0.21037 (13)	0.35509 (7)	0.85604 (5)	0.02010 (19)	
O1	0.5170 (3)	0.33271 (14)	0.79415 (13)	0.0267 (4)	
O2	0.7174 (3)	0.21637 (14)	0.70818 (13)	0.0317 (4)	
C1	0.5540 (4)	0.2377 (2)	0.75825 (17)	0.0236 (5)	
C2	0.3907 (4)	0.1430 (2)	0.77760 (17)	0.0252 (5)	
H2A	0.4712	0.0786	0.8028	0.030*	
O3	0.2403 (3)	0.18383 (14)	0.84555 (13)	0.0295 (4)	
H3D	0.146 (4)	0.1355 (19)	0.860 (2)	0.035*	
C3	0.2705 (5)	0.1069 (2)	0.6913 (2)	0.0358 (7)	
H3A	0.1694	0.0474	0.7059	0.054*	
H3B	0.3756	0.0809	0.6466	0.054*	
H3C	0.1901	0.1695	0.6665	0.054*	
O4	0.3478 (3)	0.36013 (14)	0.98528 (11)	0.0269 (4)	
O5	0.4302 (4)	0.46441 (16)	1.10705 (14)	0.0414 (6)	
C4	0.3553 (4)	0.4525 (2)	1.02739 (18)	0.0244 (5)	
C5	0.2663 (5)	0.5563 (2)	0.97857 (17)	0.0274 (6)	
H5A	0.3796	0.6150	0.9800	0.033*	
O6	0.2256 (3)	0.52574 (13)	0.88475 (12)	0.0253 (4)	
H6D	0.236 (5)	0.5821 (15)	0.8526 (16)	0.030*	
C6	0.0595 (6)	0.6001 (3)	1.0249 (2)	0.0517 (9)	
H6A	0.0080	0.6655	0.9929	0.078*	
H6B	0.0922	0.6192	1.0876	0.078*	
H6C	−0.0528	0.5432	1.0235	0.078*	
O7	0.0543 (3)	0.37074 (16)	0.73217 (12)	0.0292 (4)	
H7A	−0.052 (3)	0.3289 (19)	0.7204 (19)	0.035*	
H7B	0.062 (5)	0.4195 (17)	0.6915 (15)	0.035*	
O8	−0.0981 (3)	0.33681 (17)	0.91682 (12)	0.0295 (4)	
H8A	−0.210 (3)	0.346 (2)	0.8848 (17)	0.035*	
H8B	−0.124 (5)	0.2826 (17)	0.9512 (16)	0.035*	

### Atomic displacement parameters (Å^2^)

**Table:**

	*U*^11^	*U*^22^	*U*^33^	*U*^12^	*U*^13^	*U*^23^
Mg	0.0207 (4)	0.0195 (4)	0.0200 (4)	−0.0006 (4)	−0.0010 (3)	0.0003 (3)
O1	0.0226 (8)	0.0243 (9)	0.0332 (9)	−0.0025 (8)	0.0032 (8)	−0.0066 (8)
O2	0.0251 (9)	0.0316 (9)	0.0384 (10)	−0.0039 (9)	0.0074 (9)	−0.0109 (8)
C1	0.0212 (12)	0.0265 (12)	0.0233 (12)	0.0003 (11)	−0.0038 (10)	−0.0031 (10)
C2	0.0240 (12)	0.0205 (11)	0.0309 (13)	0.0014 (11)	0.0016 (10)	−0.0019 (11)
O3	0.0334 (10)	0.0215 (8)	0.0335 (10)	−0.0048 (8)	0.0100 (9)	−0.0012 (7)
C3	0.0281 (14)	0.0378 (14)	0.0414 (16)	−0.0077 (13)	0.0019 (13)	−0.0149 (13)
O4	0.0388 (10)	0.0197 (8)	0.0222 (8)	0.0045 (9)	−0.0099 (8)	−0.0029 (7)
O5	0.0630 (14)	0.0323 (10)	0.0288 (10)	0.0164 (11)	−0.0218 (11)	−0.0107 (9)
C4	0.0255 (12)	0.0252 (12)	0.0225 (12)	0.0035 (11)	−0.0037 (10)	−0.0014 (10)
C5	0.0384 (14)	0.0197 (11)	0.0242 (12)	−0.0004 (12)	−0.0060 (12)	−0.0035 (9)
O6	0.0352 (10)	0.0178 (8)	0.0231 (9)	−0.0014 (9)	−0.0034 (8)	0.0035 (7)
C6	0.063 (2)	0.057 (2)	0.0353 (16)	0.0379 (19)	−0.0036 (17)	−0.0096 (15)
O7	0.0322 (10)	0.0312 (10)	0.0240 (9)	−0.0088 (9)	−0.0071 (8)	0.0086 (8)
O8	0.0257 (9)	0.0354 (10)	0.0275 (10)	−0.0033 (9)	0.0014 (8)	0.0076 (8)

### Geometric parameters (Å, º)

**Table:**

Mg—O7	2.0407 (19)	C3—H3C	0.9600
Mg—O4	2.0544 (18)	O4—C4	1.261 (3)
Mg—O3	2.0549 (19)	O5—C4	1.251 (3)
Mg—O8	2.077 (2)	C4—C5	1.524 (3)
Mg—O6	2.0784 (18)	C5—O6	1.432 (3)
Mg—O1	2.079 (2)	C5—C6	1.514 (4)
O1—C1	1.267 (3)	C5—H5A	0.9800
O2—C1	1.254 (3)	O6—H6D	0.821 (10)
C1—C2	1.526 (4)	C6—H6A	0.9600
C2—O3	1.428 (3)	C6—H6B	0.9600
C2—C3	1.512 (4)	C6—H6C	0.9600
C2—H2A	0.9800	O7—H7A	0.832 (10)
O3—H3D	0.836 (10)	O7—H7B	0.830 (10)
C3—H3A	0.9600	O8—H8A	0.828 (10)
C3—H3B	0.9600	O8—H8B	0.832 (10)
			
O7—Mg—O4	172.11 (8)	H3A—C3—H3B	109.5
O7—Mg—O3	93.80 (8)	C2—C3—H3C	109.5
O4—Mg—O3	93.50 (8)	H3A—C3—H3C	109.5
O7—Mg—O8	88.18 (8)	H3B—C3—H3C	109.5
O4—Mg—O8	88.77 (8)	C4—O4—Mg	118.91 (16)
O3—Mg—O8	90.37 (8)	O5—C4—O4	124.1 (2)
O7—Mg—O6	96.20 (8)	O5—C4—C5	117.8 (2)
O4—Mg—O6	76.70 (7)	O4—C4—C5	118.1 (2)
O3—Mg—O6	169.46 (9)	O6—C5—C6	111.6 (2)
O8—Mg—O6	93.28 (9)	O6—C5—C4	107.29 (19)
O7—Mg—O1	92.51 (8)	C6—C5—C4	111.4 (2)
O4—Mg—O1	92.14 (8)	O6—C5—H5A	108.8
O3—Mg—O1	76.23 (7)	C6—C5—H5A	108.8
O8—Mg—O1	166.60 (8)	C4—C5—H5A	108.8
O6—Mg—O1	99.95 (8)	C5—O6—Mg	116.59 (13)
C1—O1—Mg	116.77 (16)	C5—O6—H6D	109 (2)
O2—C1—O1	124.0 (2)	Mg—O6—H6D	134 (2)
O2—C1—C2	117.9 (2)	C5—C6—H6A	109.5
O1—C1—C2	118.1 (2)	C5—C6—H6B	109.5
O3—C2—C3	111.3 (2)	H6A—C6—H6B	109.5
O3—C2—C1	106.8 (2)	C5—C6—H6C	109.5
C3—C2—C1	111.7 (2)	H6A—C6—H6C	109.5
O3—C2—H2A	109.0	H6B—C6—H6C	109.5
C3—C2—H2A	109.0	Mg—O7—H7A	119.0 (19)
C1—C2—H2A	109.0	Mg—O7—H7B	131.6 (19)
C2—O3—Mg	116.46 (15)	H7A—O7—H7B	109 (2)
C2—O3—H3D	112 (2)	Mg—O8—H8A	118.8 (19)
Mg—O3—H3D	127 (2)	Mg—O8—H8B	121 (2)
C2—C3—H3A	109.5	H8A—O8—H8B	106 (2)
C2—C3—H3B	109.5		

### Hydrogen-bond geometry (Å, º)

**Table:**

*D*—H···*A*	*D*—H	H···*A*	*D*···*A*	*D*—H···*A*
O3—H3*D*···O5^i^	0.84	1.83	2.668 (2)	178
O6—H6*D*···O2^ii^	0.82	1.85	2.666 (2)	174
O7—H7*A*···O2^iii^	0.83	1.94	2.768 (2)	171
O7—H7*B*···O5^iv^	0.83	1.85	2.678 (2)	177
O8—H8*A*···O1^iii^	0.83	2.12	2.933 (2)	167
O8—H8*B*···O4^i^	0.83	1.94	2.765 (2)	168

Symmetry codes: (i) *x*−1/2, −*y*+1/2, −*z*+2; (ii) −*x*+1, *y*+1/2, −*z*+3/2; (iii) *x*−1, *y*, *z*; (iv) −*x*+1/2, −*y*+1, *z*−1/2.
